# Multi-directional neutron dark-field imaging with single absorption grating

**DOI:** 10.1038/s41598-023-42310-y

**Published:** 2023-09-15

**Authors:** Matteo Busi, Jiazhou Shen, Michael Bacak, Marie Christine Zdora, Jan Čapek, Jacopo Valsecchi, Markus Strobl

**Affiliations:** 1 Laboratory for Neutron Scattering and Imaging, Paul Scherrer Institut, 5232 Villigen, Switzerland; 2 European Organization for Nuclear Research, CERN, 1211 Geneva, Switzerland; 3 Institute for Biomedical Engineering, ETH Zürich, 8092 Zurich, Switzerland; 4 Laboratory for Macromolecules and Bioimaging, Paul Scherrer Institut, 5232 Villigen, Switzerland

**Keywords:** Materials science, Physics

## Abstract

Neutron dark-field imaging is a powerful technique for investigating the microstructural properties of materials through high-resolution full-field mapping of small-angle scattering. However, conventional neutron dark-field imaging utilizing Talbot–Lau interferometers is limited to probing only one scattering direction at a time. Here, we introduce a novel multi-directional neutron dark-field imaging approach that utilizes a single absorption grating with a two-dimensional pattern to simultaneously probe multiple scattering directions. The method is demonstrated to successfully resolve fiber orientations in a carbon compound material as well as the complex morphology of the transformed martensitic phase in additively manufactured stainless steel dogbone samples after mechanical deformation. The latter results reveal a preferential alignment of transformed domains parallel to the load direction, which is verified by EBSD. The measured real-space correlation functions are in good agreement with those extracted from the EBSD map. Our results demonstrate that multi-directional neutron dark-field imaging is overcoming significant limitations of conventional neutron dark-field imaging in assessing complex heterogeneous anisotropic microstructures and providing quantitative structural information on multiple length scales.

## Introduction

Neutron dark-field contrast imaging^1^ has proven to be a powerful analytic tool for assessing microstructures, and in particular variations of microstructure in bulk materials and devices, due to its unique combination of concurrently observed scales. It combines direct real-space information of neutron imaging on the scale of tens of micrometers to centimeters with pixel-wise small-angle scattering information on the tens of nanometers to micrometer scale^2–4^. Advanced quantitative neutron dark-field imaging offers not only qualitative insights regarding structural inhomogeneities within sample volumes but also quantitative information on the respective local microstructures by providing real-space correlation functions, which are measured e.g. through sample-detector distance scans^5^. Therefore, this technique not only provides new understandings of the microstructural properties of materials but also offers a promising avenue for non-destructively investigating complex structures in bulk materials and assemblies subjected to external and internal stimuli, both in radiographic mode providing 2D thickness-average maps, and in tomographic mode, offering volumetric bulk insights of the studied materials^6^. Prominent examples of applications include the visualization of magnetic domains in electrical steels^7–9^, including time-resolved studies allowing to quantify the movement of domain walls in grain oriented electrical steels^10^. This technique also has potential in characterizing porosity, cracks and phases in engineering materials including additively manufactured metals^11–13^. Additionally, it can be used to study water distribution in gas diffusion layers of fuel cells^14^, as well as for soft-matter investigations^5,15,16^.

Neutron dark-field imaging^1^ was first enabled through Talbot–Lau neutron grating interferometry (NGI)^17^. Analyses rely on the evaluation of the sample-induced damping of a spatial beam modulation, which is established through interference effects that are generated and probed by a set of three line gratings. Small-angle scattering from the local microstructure in a sample leads to a local reduction of the visibility of the intensity modulation. Since the full period of the interference pattern is typically smaller than the effective pixel size of the detector system, it cannot be resolved directly. Therefore, a phase-stepping procedure of one of the gratings is used to evaluate the pattern within each pixel of the imaging detector. Corresponding phase-stepping curves without (reference) and with the sample are acquired, and the ratio of the extracted pattern visibilities constitutes the dark-field signal. However, neutron dark-field imaging with a Talbot–Lau grating interferometer is limited in its ability to probe anisotropic scattering due to the directional characteristic of the line gratings employed and the corresponding phase-stepping approach. Thus, only one scattering direction, perpendicular to the grating lines, can be probed without changes to the experimental setup, such as rotating the sample or the gratings.

A recent study has established the feasibility to overcome the uni-directional sensitivity limitation of Talbot–Lau neutron dark-field imaging^18^. It has been shown that oriented microstructures with anisotropic scattering can be probed using a multi-directional grating interferometer employing a set of circular gratings that allow the simultaneous acquisition of dark-field images with respect to multiple scattering directions. However, the multi-directional nature hinders the utilization of a phase stepping analysis, and thus relies on modulation periods resolvable by the detector system. In this regime, characterized by modulation periods of the order of hundreds of micrometers, another dark-field imaging approach has been realized recently. A beam modulation induced by a single absorption grating has been shown to be effective for dark-field contrast imaging^19^. Single grating approaches have also been explored with X-rays^20–22^ and a recent paper describes the utilization of an orthogonal 2D pattern for directional dark-field contrast imaging^23^. Such an approach overcomes all complexity of alignment and in particular it is achromatic, in contrast to diffractive phase grating methods. This makes the technique also highly compatible with time-of-flight measurements. The achievable modulation visibility, in this case, for a certain distance from the detector depends on the pinhole collimation of the beam and thus impacts the available intensity. Adding a source grating could overcome this limitation, however this would add back complexity to the setup. The distance from the detector limits the longest probed correlation length that can be probed, while a larger distance increases the requirements on collimation. However, it has been shown recently by Busi et al.^24^ that beyond the visibility minimum based on the geometrical resolution limit an inverse modulation pattern gains visibility and can equally be exploited for dark-field contrast imaging. This effect can increase the probable autocorrelation range dramatically by enabling increased sample-to-detector distances. Remarkably, even further alternating positive and inverse visibility peaks can be found, however with decaying amplitude.

Here, we present a novel multi-directional neutron dark-field imaging approach based on the single absorption grating technique utilizing two-dimensional patterns to probe multiple scattering directions simultaneously, while eliminating the limitations and complexity of phase gratings. We show that also two-dimensional grating patterns enable exploiting the inverse pattern modality to extend the autocorrelation length range probed. To validate the method, we record and analyze the directional dark-field signals of carbon fiber samples, i.e. strongly aligned artificial anisotropic microstructures at multiple well known orientations. We then demonstrate the potential of the method on a more complex sample by investigating the microstructure of additively manufactured stainless steel AISI 304L samples and in particular the anisotropic morphology of the martensite induced by mechanical deformation.

## Materials and methods

The method used to obtain transmission (T), differential phase (DP), and dark-field (DF) maps from the recorded intensity-modulated images, builds upon the spatial harmonic analysis^20–22^, which analyzes discrete peaks in the spatial frequency domain obtained through a 2D Fourier transform of the raw image. However, differently from previous studies, we introduce an analyzing window $\mathscr {A}$ defined as

1 $$\begin{aligned} \mathscr {A}(x,y) = I(\left[ x-w,x+w\right] ,\left[ y-w,y+w\right] ), \end{aligned}$$

where $(2w+1)$ is the window size along one axis, and *x* and *y* are the image pixel coordinates. For each pixel with coordinates (*x*, *y*), a 2D-Fourier transformation is applied to the analyzing window $\mathscr {A}$ and the result is transformed into polar coordinates, $\mathscr {A}_\mathscr {F}(r,\theta )$, with $x=r\cos \theta$ and $y=r\sin \theta$. The first harmonic coefficient, *a*, of the Fourier transform can be obtained by evaluating $\mathscr {A}_\mathscr {F}$ at $r=0$. The transmission can then be calculated as

2 $$\begin{aligned} \textrm{T}=a/a_0, \end{aligned}$$

where the index 0 always refer to the undisturbed open-beam measurement (with only the single grating in the beam). The second harmonic coefficients, $b(\theta ) = \mathscr {A}_\mathscr {F}(r=r_b,\theta )$, are located at angles $\theta$ determined by the directional periodicity of the absorption grating and at a radius $r_b$ dependent on the modulation period *p* along the corresponding angle. The absorption grating visibility, V, in the direction $\theta$, can be computed as:

3 $$\begin{aligned} \textrm{V}(\theta )=\frac{|b(\theta )|}{a}, \end{aligned}$$

which allows for the calculation of the directional dark-field signal

4 $$\begin{aligned} \textrm{DF}(\theta )=\frac{\textrm{V}(\theta )}{\textrm{V}_0(\theta )}. \end{aligned}$$

Finally, the differential phase contrast can be calculated as:

5 $$\begin{aligned} \textrm{DP}(\theta )=-\arg (b(\theta )+\textrm{i}b(\theta )) + \arg (b_0(\theta )+\textrm{i}b_0(\theta )). \end{aligned}$$

Although the technique enables obtaining all three imaging modalities simultaneously, this work focuses particularly on the dark-field contrast as it is most relevant for the microstructural material characterization considered. It is noted that the DF and DP contrast expressed in Eqs. 4, 5 are well-determined when the visibility is high enough, i.e. when the second harmonic peaks are dominant with respect to the background in the Fourier domain, indicating that the intensity modulation can be effectively represented by angular dependent sinusoidal functions.

**Figure 1:**
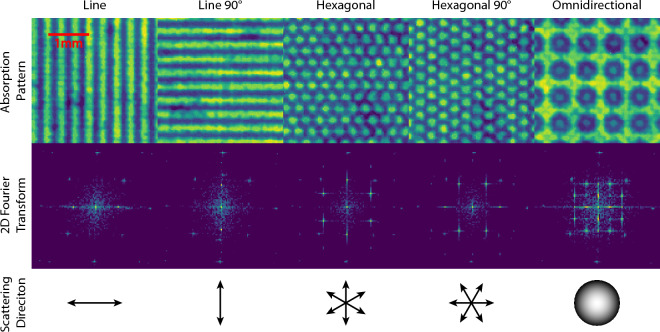
Direct transmission contrast radiographs of the single absorption gratings. Line, hexagonal and omnidirectional patterns are shown, as well as 90$^\circ$ rotations of the line and hexagonal patterns. The 2D Fourier transform images respective to the patterns exhibit their first harmonic peak in the central pixel and second harmonic peaks radially distributed in number and angles depending on the structure of the pattern. The scattering direction sensitivity resulting from the patterns is shown in the last row.

The radiographs of various absorption grating patterns, along with their corresponding 2D Fourier transform and scattering direction sensitivity, are presented in Fig. 1. The figure displays outcomes for three distinct grating designs: (1) a conventional *line* pattern, which is only sensitive to a single scattering direction, (2) a *hexagonal* pattern, which is sensitive to three scattering directions, and (3) an *omnidirectional* pattern, which is theoretically sensitive to all transversal directions. However, in reality also the latter is limited to a specific number of directions dependent on the pattern’s resolution as a discrete pixelated image. Measurements were also conducted on the line and hexagonal patterns after rotating them by 90$^\circ$, which in both cases provides access to additional scattering directions. For the line grating, the one-fold periodicity of the pattern results in two distinct second harmonic peaks in the 2D Fourier transform, which are aligned with the direction of scattering sensitivity and rotate accordingly as the grating is rotated (compare Line and Line 90$^\circ$). Similarly, the hexagonal grating produces six second harmonic peaks in the 2D Fourier transform due to its pattern design. It is worth noting that although all the peaks in the Fourier domain can be utilized to determine the DP contrast images across a range of [0, 2$\uppi$], the modulus operator in Eq. 3 imposes a $\uppi$ symmetry on the peaks in Fourier space, which results in a sensitivity range of [0, $\uppi$] for DF contrast images. This is in accordance with the symmetry of small-angle neutron scattering and one of the basic conditions for DF contrast imaging to provide quantitative meaningful results^2^.

It is worth noting that for the method described here the visibility is primarily dependent on the absorption efficiency of the grating pattern and the imaging system’s ability to resolve it spatially. This is largely determined by the size of the pinhole used to collimate the beam and the distance between the grating and the imaging detector $L_{\textrm{G}}$. The latter parameter determines the range of autocorrelation lengths^2^

6 $$\begin{aligned} \xi = \frac{L_{\textrm{S}} \lambda }{p} \end{aligned}$$

that can be probed with the setup, where the wavelength $\lambda$, or at least the maximum and minimum accessible wavelengths, as well as the utilized grating period *p* are fixed, and the sample needs to be positioned between the grating and the imaging detector at a distance $L_S$ from the latter, which is limited according to $L_{\textrm{G}}>L_{\textrm{S}}>0$.

## Experiments and results

The single absorption grating setup for multi-directional DF imaging was installed at the ICON beamline^25^ located at the SINQ neutron source of the Paul Scherrer Institut (PSI), Switzerland. The setup consisted of a 100 μm-thick scintillation screen made of LiF and ZnS(Ag), the light of which is recorded by an Andor iKon-L CCD camera (2048 $\times$ 2048 pixels$^2$) with a 100 mm Zeiss f/2 objective lens. The lens magnification was adjusted to obtain an effective pixel size of 35 μm and a field of view of 72$\times$72 mm$^2$. The absorption grating was composed of a 20 μm-thick gadolinium layer, with a size of 70$\times$70 mm$^2$, deposited on a 300 μm-thick round silicon wafer. The desired grating pattern was achieved by laser ablation of the gadolinium coating (KLUG Laserdienstleistungen und Beratung, Hannover). The experiments presented below utilized only the *hexagonal* pattern grating, as shown in Fig. 1. This grating comprises 175 μm-diameter round holes arranged in a honeycomb hexagonal pattern with a period of 350 μm. The grating was positioned at a distance of $L_{\textrm{G}}= 24$ cm upstream of the detector, precisely at the second visibility maximum of the grating pattern, which is in the inverse pattern regime^24^. This positioning allowed for a wider range of autocorrelation lengths to be probed compared to the direct pattern regime. The grating visibility ($V_0(\theta )$) obtained was approximately 0.11 for the three scattering directions probed using the hexagonal grating. Taking into account the mean wavelength 2.8 ${ \text{\AA }}$ of the beam spectrum at ICON and the grating period, the probable autocorrelation lengths ranged between a few tens and 200 nanometers, where the lower boundary is determined by the closest achievable proximity of the sample to the grating (Eq. 6). The acquisition procedure consisted of 11 exposures of 1 min for each image, of which a median image was extracted, to eliminate outliers originated from e.g. gamma background.

The first sample measured in this study consisted of four specimens made of carbon fibers, which were arranged in different directions with regards to the highly aligned fiber microstructure. The fiber samples, which were chosen to demonstrate the resolution and assess the orientation of the microstructures, were mounted on a small aluminum plate, which is only visible in the transmission image.

**Figure 2:**
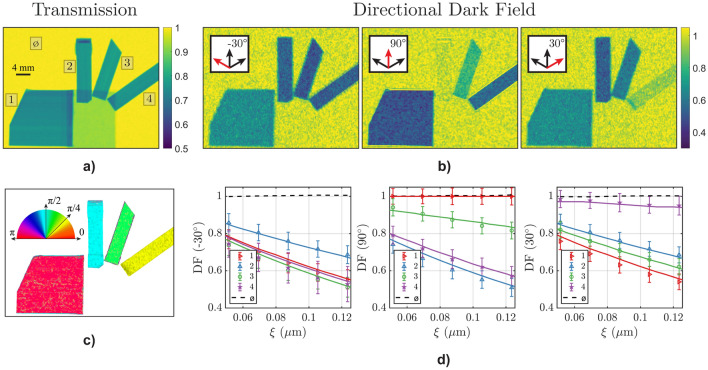
(**a**) Transmission contrast image of the carbon fiber samples (attached to an aluminum plate). (**b**) Directional dark-field contrast images of the carbon fibers samples using the “Hexagonal” pattern grating shown in Fig. 1. The scattering direction probed in the corresponding graphs is shown as red arrow in the insets. (**c**) Microstructural orientation map of the carbon fibers. (**d**) Autocorrelation length curves of the four carbon fiber samples and the background (black-dashed line), for the three probed scattering directions. The solid lines are the theoretical curves, obtained from calculations with the known structural parameters, whereas the markers are the measured values in the respective regions-of-interest with corresponding error bars.

Figure 2a,b depict the transmission and directional dark-field (DF) contrast images obtained using the *hexagonal* grating for three different scattering directions namely − 30$^\circ$, 30$^\circ$ and 90$^\circ$. The carbon-fiber samples analyzed in this study are labeled 1, 2, 3, and 4, and their fiber orientations are 0$^\circ$, 90$^\circ$, 110$^\circ$, and 135$^\circ$, respectively. In the transmission contrast image, all four samples display the same contrast since they have the same thickness and the same attenuation coefficient. Only the contrast between the carbon fibers and the aluminum plate utilized to mount them is visible. An imperfection regarding the thickness of sample 2 is observed at the top edge. In contrast, the directional DF images provide significant differences in contrast for the carbon-fiber samples, which depends on the relative angle between the fibers and the probed scattering direction. For instance, in the 90$^\circ$ DF image, sample 2 appears nearly transparent since the carbon fibers are aligned with the probed scattering direction. Conversely, sample 1, which has carbon fibers aligned perpendicular to the probed scattering direction, displays the highest contrast in the same image. Similar angular dependencies are observed for the other samples and are illustrated in Fig. 2d, which depicts the average DF contrast in a region of interest, as a function of the autocorrelation length, measured by altering the distance $L_{\textrm{S}}$ between the sample and the detector.

To validate the experimental findings, we have calculated theoretical DF curves using the general equation for quantitative DF contrast analyses of correlation length scans^2^:

7 $$\begin{aligned} DF(\xi ,\theta ) = \exp \left( {\lambda ^2 (\Delta \rho )^2 \phi (1-\phi)\chi t (G(\xi ,\omega )-1)}\right) , \end{aligned}$$

where $\Delta \rho$ is the scattering length density (SLD), $\phi$ is the volume fraction, $\chi$ is the length scale of the structures observed and *t* is the sample thickness along the neutron beam. To model the carbon fibers, tubular particles with an infinite length, a diameter of 5 μm, a $\Delta \rho$ value of 4.3 $\times$ 10$^{-6}$ Å^−2^, a volume fraction $\phi$ of 0.50, and a sample thickness of 1 mm were simulated. The two-dimensional autocorrelation function of such a system was calculated for the simulated structure and transformed into polar coordinates to obtain the angular-dependent correlation function G$(\xi ,\omega )$, where $\omega$ is the angle between the probed scattering direction $\theta$ and the long axis of the fibers. The experimental results demonstrate good agreement with the model across all carbon fiber orientations and probed scattering directions (see Fig. 2d). This agreement enabled us to accurately determine the microstructural orientation of the carbon fibers from the experimental data, by fitting the directional dark-field contrast to the theoretical model. Figure 2c depicts the orientation map of the four samples, where the areas without dark-field contrast have been masked out. The map was generated by minimizing the average root mean square error between the directional dark field signal in each pixel and the theoretical curves as a function of the microstructural direction.

**Figure 3:**
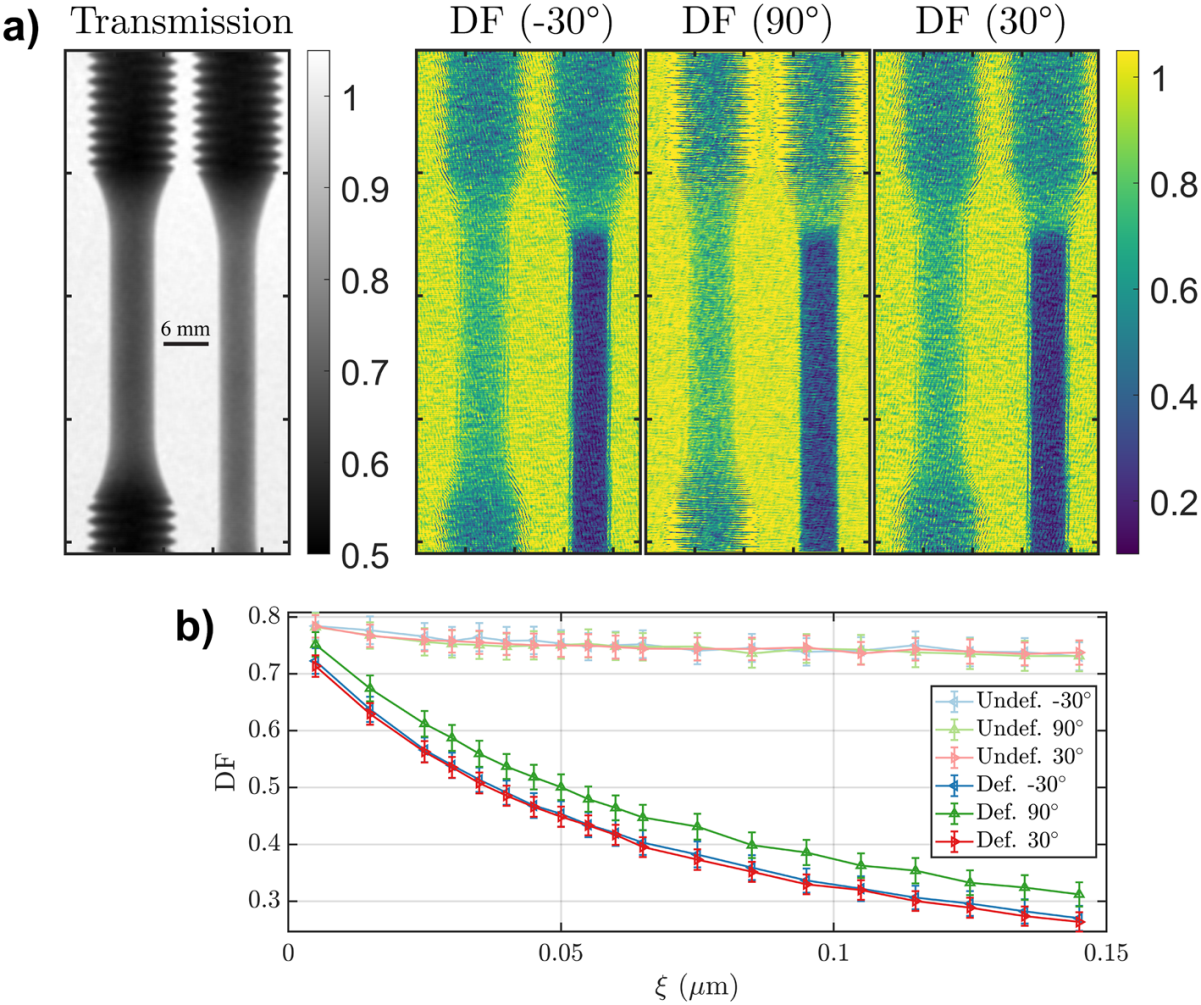
(**a**) Transmission and directional dark-field (at the largest autocorrelation length probed) contrast maps of the two round dogbones stainless steel samples. (**b**) Average region-of-interest dark-field signal as function of the correlation length and the probed scattering directions, for the deformed and undeformed sample.

Subsequently, a set of round dogbones made of AISI 304L stainless steel were measured. The set consisted of one unaltered specimen (as fabricated), and one specimen that had undergone uniaxial tensile loading. The samples are the same type as used in a previous study using conventional Talbot–Lau (cTL) neutron dark-field imaging to characterize microstructural properties such as porosity and build defects, and which found substantial dark-field contrast caused by a partial crystallographic phase transformation from austenite (FCC) to martensite (BCC) caused by tensile load deformation^13^. While this study was successful in quantifying the respective transformed phase fraction, the morphology of the transformed phase partitions could not be elucidated. Figure 3 displays the transmission and DF maps obtained for the two samples in this study. The deformed sample can easily be identified in the transmission contrast image, as it is elongated and thinned. But apart from the different thickness the overall contrast between the two samples is negligible.

**Figure 4:**
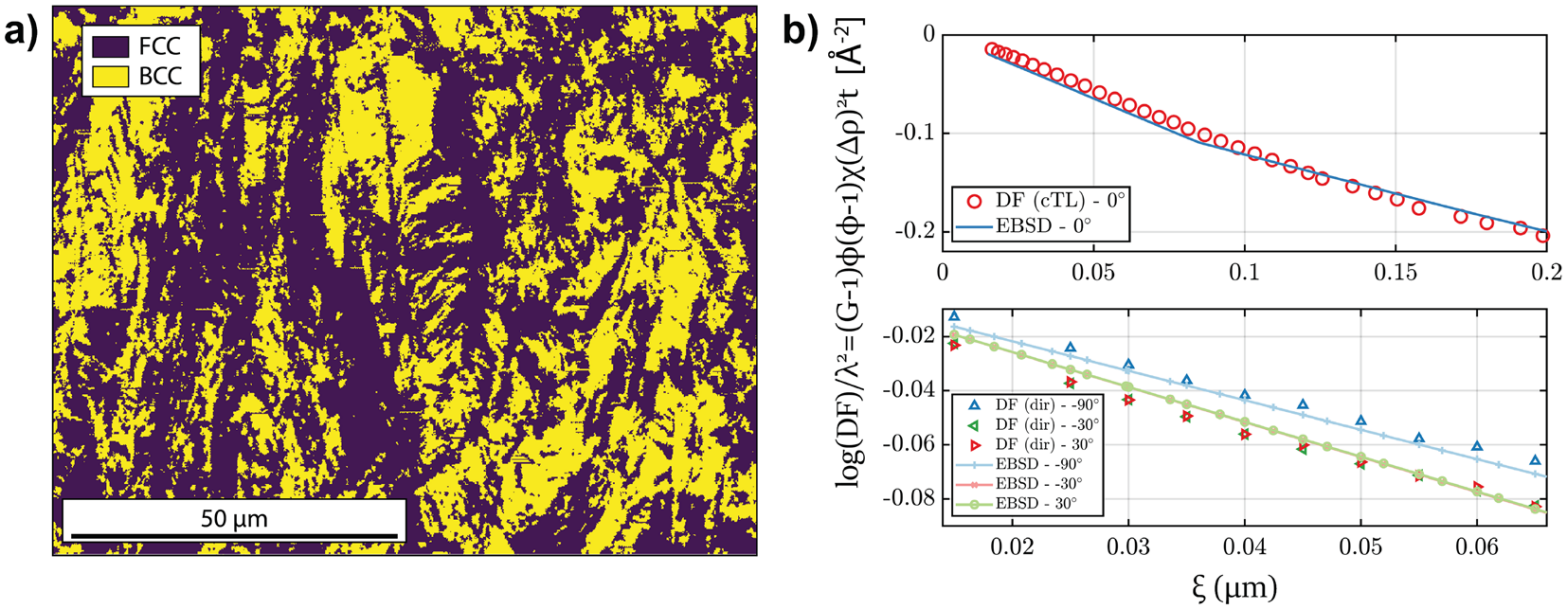
(**a**) EBSD map segmentation of austenitic (FCC) and martensitic (BCC) phases in the deformed dogbone sample. (**b**) Top: Comparison between experimental DF using conventional Talbot–Lau (cTL) grating interferometry (data from^13^) and a curve calculated from the EBSD map projected to the horizontal scattering direction. Bottom: Comparison between directional correlation functions extracted from the multi-directional DF imaging experiment and corresponding curves derived from EBSD measurements.

The DF map, on the other hand, shows a faint image of the samples, in areas where no transformation has taken place. This remaining DF contrast is most likely due to nano-porosity and potential minor amounts of precipitates^26^. In contrast, the deformed region displays a remarkable dark-field contrast, which can be attributed to the presence of martensite that has transformed from the austenitic steel matrix. While the dark-field signal of the undeformed sample displays no anisotropy with regards to the three probed scattering directions, as can be seen in the plots of dark-field contrast versus probed correlation length (Fig. 3b), the deformed sample region reveals clearly different scattering for different directions. A symmetrical anisotropy with respect to the tensile load direction (90$^\circ$) can be observed. It can be attributed to a preferential orientation of the martensitic microstructure parallel to the load direction. As a result, the dark-field signal parallel to the load direction is weaker than for the other two directions, which are symmetrical at − 30$^\circ$ and 30$^\circ$. In order to validate these findings, electron backscattered diffraction (EBSD) was applied in one position of the deformed sample to obtain a segmentation of the austenitic (FCC) and martensitic (BCC) microstructure (Fig. 4a).

To perform a quantitative comparison with the measured DF curves for the martensitic microstructure in the deformed stainless steel sample, we calculated the autocorrelation map of the martensitic structure in the segmented EBSD map. This way, an angular-dependent correlation function was derived. Utilizing the previously reported value of $\Delta \rho ^2 \phi (1-\phi)$ = 3.5 $\times$ 10$^{-14}$ ${ \text{\AA }}^{-4}$^13^ and the sample thickness of 6 mm for calculating the theoretical dark-field curves from the correlation function a very good agreement with the experimental results is found for all probed directions (Fig. 4). Thus, we confirmed that the quantitative nature of the multidirectional dark-field contrast analysis could be verified, paving the way to numerous applications in assessing heterogeneous anisotropic microstructures in a wide range of materials.

## Conclusions

We have introduced a novel neutron imaging technique capable of acquiring multi-directional dark-field, differential phase, and conventional transmission contrast maps simultaneously using a single absorption grating. The method significantly simplifies the measurement of anisotropic structures using only one absorption grating, allowing for the sensitivity to multiple scattering directions to be tuned by its design. Additionally, this method can be effectively integrated with a time-of-flight approach since the grating’s visibility remains constant across different wavelengths, making it achromatic. We demonstrated the feasibility and capability of the technique to perform quantitative characterization using carbon fiber samples, the orientation and structure of which could be extracted. This constitutes the potential to analyze fiber orientations and strongly aligned microstructures in bulk materials with multi-directional dark-field imaging. Furthermore, the technique was successfully applied to reveal and describe the complex morphology of a transformed crystallographic phase in an additively manufactured stainless steel sample in terms of an anisotropic structural correlation function measured directly with multi-directional dark-field contrast imaging. It was found that the resulting microstructure exhibits a preferential alignment with the load direction. Thus, the experimental findings demonstrated the capability to not only determine distinct microstructure orientation without rotating either the sample or the gratings, but also complex anisotropic structural correlations. This could be verified by the application of a conventional local EBSD measurement. It can therefore be concluded that the introduced multi-directional dark-field imaging method is well suited for quantitative two-dimensional small-angle scattering analysis of heterogeneous samples with structures in the nanometer range, where conventional methods, averaging over a large volume, might fail. The method offers microstructure resolution on length scales ranging from a few to a few hundreds of nanometers paired with direct spatial resolution on the order of hundred micrometers. Potential applications are considered in fields such as soft matter polymer science and rheological studies, additive manufacturing, but also for the investigation of magnetic structures and phenomena.

## Data Availability

The datasets used and/or analysed during the current study available from the corresponding author on reasonable request.
